# Dental Caries Pattern and Treatment Needs among Ugandan Adolescent Students: A Cross-Sectional Study

**DOI:** 10.1155/2020/8135865

**Published:** 2020-03-09

**Authors:** Barbara Ndagire, Catherine L. Mwesigwa, Juliet M. Ntuulo, Harriet Mayanja-Kizza, Damalie Nakanjako, Charles M. Rwenyonyi

**Affiliations:** ^1^Department of Dentistry, College of Health Sciences, Makerere University, Kampala, Uganda; ^2^Department of Internal Medicine, School of Medicine, College of Health Sciences, Makerere University, Kampala, Uganda

## Abstract

Dental caries is still a major public health problem owing to its high prevalence and incidence in several regions. Planning and development of effective preventive and treatment modalities for the management of dental caries demand information on disease pattern and treatment needs of the populations. However, there is a paucity of this information in Uganda. The aim of the present study was to identify the dental caries pattern and treatment needs among Ugandan adolescent students. This was a descriptive cross-sectional study conducted among 11- to 19-year-old adolescents attending two secondary schools in Kampala and Mukono districts of Uganda. At both schools, random sampling was used to select the participating classes and the adolescents. Decayed teeth and treatment needs were recorded using the World Health Organization Basic Oral Health Survey criteria. A total of 406 adolescents comprising of 249 female and 157 male students participated in the study. Data were analysed using STATA, version 12.0. The prevalence of decayed teeth (DT) was expressed as a percentage of individuals with DT score ≥1. The treatment needs were categorised into three groups. Associations between dependent and independent variables were evaluated using cross-tabulation, chi-square test, and Poisson regression analysis. The overall prevalence of decayed teeth was 62.6% and mean DT was 1.7 ± 2.3. A total of 696 decayed teeth were observed, and the molar teeth, particularly the second molar (50.6%), were the most significantly affected. The prevalence of caries was higher in the mandible (51.4%) compared to the maxilla though the difference was not statistically significant. Decayed teeth were significantly (*p* < 0.05) associated with difficulty in chewing, history of dental pain in the past 12 months, poor perception of tooth state, and the female participants. Majority (59.4%) of the study participants required restorations of teeth. About 83.2% (*n* = 579) of the teeth needed restorations, while 44 needed extractions. In conclusion, the prevalence of decayed teeth was high among the study population. It is recommended that school health programmes should include oral health preventive and curative interventions to achieve optimum health.

## 1. Introduction

Worldwide, dental caries and periodontal diseases still constitute a major public health problem due to their high prevalence and significant impact on general health [1, 2]. In Africa, previous reports indicated that the prevalence and severity of dental caries have been low [3, 4]. However, there is now evidence of a rise in the prevalence of dental caries in some countries [5, 6]. The rise has been attributed to the growing consumption of refined sugars and inadequate use of fluoride [3, 7]. An increase in the prevalence of dental caries in the developing countries including Uganda places a bigger burden on the limited resources available for oral health [8]. Oral healthcare services in Uganda are provided at both government and private facilities by two cadres: the public health dental officers and dentists [9]. According to the Ministry of Health, basic oral health services are supposed to be free in government health units. However, due to gross shortages of materials, supplies, equipment, and manpower, patients have to seek and pay for the basic treatment elsewhere [9]. Combined with lack of a universal health coverage insurance scheme in Uganda today, the burden of financing health services is often borne by patients [10]. Decayed teeth lead to pain, loss of school hours, and diminished general health and quality of life of the affected individuals [3, 11, 12]. In Africa, data on dental caries pattern and treatment needs are scarce. Much of the available information focuses on epidemiological data with respect to the incidence and prevalence of dental caries in specific populations. Additionally, Uganda has limited oral health data for the adolescents, though they constitute a substantial percentage (26%) of the population [13]. Adolescence is a critical age important in oral health prevention because many oral health behaviours, beliefs, and attitudes develop during this period [14].

In order to counteract the potential increase in the prevalence of dental caries in developing countries and to meet 2020 global targets of the World Health Organization (WHO)/International Dental Federation (FDI) [15], there is need to have current epidemiological data depicting the disease pattern and treatment needs for specific populations [16, 17]. Such data are important for the effective planning and design of dental services, as well as choosing interventions appropriate to the local circumstances targeting those at great risk [16–18]. Without such evidence on the population needs, there arises the danger of a top-down approach in providing health services [19].

The aim of the present study was to identify the pattern and treatment needs of dental caries among Ugandan adolescents attending secondary schools.

## 2. Materials and Methods

### 2.1. Study Design and Sample Selection

This was a cross-sectional study conducted in February and March 2018 in two secondary schools in Uganda. The schools are located in the urban setting of the two districts: Kampala and Mukono in the central region of Uganda. Kampala is the capital city of Uganda and the most densely populated urban centre, while Mukono town is the capital town of Mukono district and is located about 23 kilometres east of Kampala city. In both towns, simple random sampling was used to select one school from the list of schools provided by the respective District Education Offices. Mukono High School was selected from Mukono town while Kitebi Secondary School was selected from Kampala city. During the study period, a total of about 3,000 and 2,035 students were enrolled in senior one to six classes in Kitebi Senior Secondary School and Mukono High School, respectively. The present study was part of a cross-sectional survey to determine the prevalence, severity, and factors associated with dental caries among school adolescents in Uganda (in print).

The sample size was calculated allowing for a 5% precision, a *Z*-statistic of 1.96 for 95% confidence interval, and an assumed dental caries prevalence of 40.2% based on earlier results of Kiwanuka and Åstrøm [20] among 10- to 14-year-old students in Kampala. The statistical sample size was estimated to be 369 students to which a 10% adjustment (*n* = 37) was added to cater for nonresponse or recording errors, resulting in a total sample of 406 students. In each school, all adolescent students were informed about the study and those aged 11 to 19 years were requested to participate. In order to obtain a uniform representation of all the six classes in the two schools, about 34 participants were randomly recruited from each class using a class list (197 in Mukono and 209 in Kampala district). Simple random sampling was used to select the 34 students using class lists. The study population of 406 adolescents consisted of 249 female and 157 male students (Table 1).

### 2.2. Data Collection

Data collection involved clinical oral examination and oral interview in the form of a questionnaire. Before the main survey, four clinical examiners were trained and calibrated in recording dental caries using the Decayed, Missing, and Filled Teeth (DMFT) index and treatment need according to WHO criteria for Basic Oral Health Surveys [16]. The calibration of the examiners was done on the detection of dental caries and treatment needs using 20 adolescents who were not part of the study population. The measurements obtained were analysed using Cohen's kappa statistics for intraexaminer and interexaminer reliability giving values ranging from 0.8 to 0.9. During the main survey, each examiner carried out duplicate examinations to assess interexaminer reproducibility on 10% (*n* = 10) of randomly selected participants, which gave Cohen's kappa values of 0.8–1.0, with no evidence of systematic error (*p* > 0.5, Wilcoxon test).

During oral examination, 2 dentists worked in pairs, one as an examiner and the other as a recorder. Examination was done under field conditions in daylight with a participant lying on a portable couch in a supine position in a tree shade in the school compound. Recording of dental caries was done using disposable gloves, plane mouth mirrors, and probes after wiping off soft food debris from teeth with gauze. The dental caries status and the treatment needs were determined using the WHO oral assessment criteria [16]. For purposes of this study, clinical data were limited to decayed tooth that was defined by the presence of a lesion in a pit/fissure or on a smooth surface with a detectable softened floor/wall, undermined enamel, or temporary filling. On proximal surfaces, the probe had to enter the lesion with certainty. When in doubt, the tooth was recorded as sound. Stained pits or fissures that caught the probe, but did not have undermined enamel, softened floor or walls were also recorded as sound. The overall treatment needs required were categorised into three types of basic needs: those relating to dental caries, traumatic dental injuries, and caries preventive treatment. The specific treatment needs for the decayed teeth were divided into three groups: category 1: filling; category 2: pulp therapy; and category 3: extraction. A decayed tooth was considered for extraction if it was severely damaged by decay that only retained roots were present or there was irreparable damage to the crown. Two trained research assistants administered a modified version of the standardised WHO Oral Health Questionnaire for adults [16]. Among other variables, the sociodemographic data, perceived state of their teeth, and oral health habits were captured. The participants were asked about the state of their teeth, and the responses were recorded on a five-point Likert scale. The responses were categorised as good (1) for excellent to good, average (2) for average, and poor (3) for original categories poor to very poor. Responses to difficulty in chewing were captured as follows: (0) “don't know,” (1) “no,” (2) “sometimes,” (3) “often,” and (4) “very often”. During analysis, they were dichotomized into (1) “no” (original categories 0 and 1) and (2) “yes” (original categories 2, 3, and 4).

### 2.3. Statistical Analyses

The data were analysed using STATA, version 12.0 (College Station TX, USA). Cohen's kappa coefficients were used to assess inter- and intraexaminer agreements. Wilcoxon signed ranks test for paired observations was used to check for intraexaminer systematic errors in recording caries. Descriptive statistics were used to summarise the data. The proportions of students with decayed teeth (DT score ≥1) and for the different treatment needs were estimated. The severity of decayed teeth was categorised as DT score = 1, DT score = 2–3, DT score = 4–6, and DT score ≥7. Proportions for the prevalence of DT within specific teeth or dental arches were compared using the two-sample test for proportions. Chi-square test and cross-tabulations were used to quantitatively analyse the relationship between dependent and independent variables. Bivariate and multivariable Poisson analyses at 5% level of significance were used to analyse the relationship between DT and independent variables. *p* < 0.05 was considered to be statistically significant.

Ethical considerations followed the guidelines of the Helsinki Declaration [21]. Prior to data collection, ethical clearance was obtained from the Makerere University School of Health Sciences Institutional Review Board (Ref. no. 2017–039) as well as Uganda National Council of Science and Technology. Permission to carry out the study was obtained from the respective districts and school authorities. Informed consent was obtained from the parents or guardians of participants aged 11–17 years and the adolescents aged ≥18 years. The adolescents aged 11–17 years also assented before participating in the study. After the survey, oral health education for the prevention and control of dental caries was given to the students.

## 3. Results

The overall mean age of the participants (*n* = 406) was 15.8 ± 2.1 years with a median of 16 years. There were 157 (38.7%) boys with a mean age of 16.0 ± 2.2 years and 249 (61.3%) girls with a mean age of 15.7 ± 2.0 years (Table 1). About 93% (379/406) of the participants reportedly brushed their teeth at least once a day. About 48% (198/406) of the students had experienced dental pain in the last 12 months while 42.3% (171/406) reported chewing problems (Table 1). Seventy-four percent of the participants' mothers had attained secondary or tertiary education.

**Figure 1:**
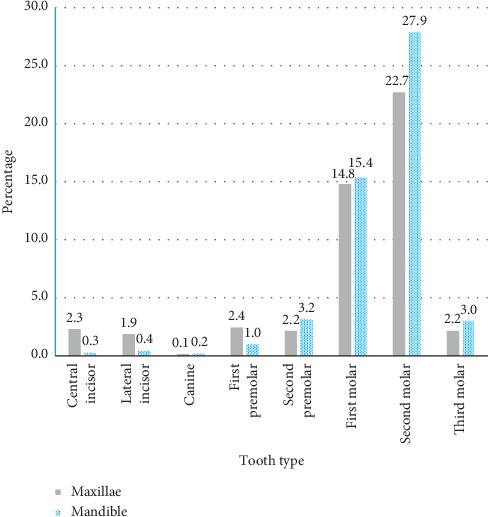
Frequency distribution of decayed teeth according to tooth types and jaws among the participants (*n* = 696 teeth).

The prevalence of decayed teeth (DT) was 62.6% (*n* = 254), and the mean DT was 1.7 ± 2.3. Majority (63.0%) of the students with a DT score > 1 had more than one decayed tooth (Table 2). Significantly more decayed teeth were recorded in female students compared to their male counterparts (*p*=0.031). However, there was no significant difference in the number of decayed teeth across gender among the students (*p*=0.806) (Table 2). The molar teeth were the most affected in both jaws while the incisors and canines were the least affected teeth (Figure 1). The second molars were significantly more affected compared to first molars (*p* < 0.001). The prevalence of caries was higher (51.4%) in the mandible than the maxilla although the difference was not statistically significant (*p*=0.460). Based on multivariate analysis, decayed teeth were significantly associated with difficulty in chewing (95% CI: 1.03–1.46, *p*=0.022), history of dental pain in the past 12 months (95% CI: 1.55–2.26, *p* < 0.001), poor perception of tooth state (95% CI: 1.17–1.82, *p*=0.001), and the female participants (95% CI: 0.71–0.97, *p*=0.017) (Table 3). More than half (59.4%) of the students needed restorative treatment, whereas 13.3% and 9.1% needed pulp therapy and extractions, respectively (Table 4). About 2.7% of the participants needed treatment for traumatic dental injuries (Table 4). Most (83.2%, *n* = 579) of the decayed teeth needed restorative treatment (Table 5). Among the 10- to 13-year-olds, most (50/55) decayed teeth needed restorative treatment while the older age groups needed more extractions or endodontic treatment (Table 5).

## 4. Discussion

The overall prevalence of decayed teeth was high. In both dental arches, the molar teeth were the most affected and decayed teeth were significantly more prevalent in the second molars. However, no significant differences in the prevalence of decayed teeth in either arch were observed in this study. The decayed teeth were found to be significantly associated with difficulty in chewing, history of dental pain in the past 12 months, poor perception of tooth state, and the female gender. Restorations were the main treatment required by the study participants.

Overall, decayed teeth were more prevalent in the mandible than the maxillae though the difference was not statistically significant. This finding is contrary to previous reports that observed significant differences between the two dental arches [22–24]. We recorded significantly more molars with decay compared to all the other teeth (Figure 1) in support of previous studies [22, 25]. The carious lesions were significantly more prevalent in the second molars compared to the first molars, which corroborates with studies from African populations [22, 26–28]. In contrast, other studies [23, 29, 30] from developing countries reported the first molars to be more frequently involved with caries compared to the second molars. Various reasons have been proposed for the differing caries susceptibilities of teeth including tooth anatomical features, eruption times, posteruptive enamel maturation of the surfaces, and the individual's age of exposure to high cariogenic diet [31, 32]. The higher susceptibly of the second molars to decay in the present study might be due to exposure of the second molar to high cariogenic diet on eruption before maturation as suggested by Loto et al. [28]. The mean age at which all the four second molars erupt ranges from 10.6 to 11.1 years in the Ugandan population [33], a critical period in which the risk for dental caries remains high especially due to adolescent diet, which is typically characterised with a high consumption of refined sugars [34, 35]. In the present study, most of the students with decayed teeth had several teeth affected. These findings are comparable to previous reports [26, 35] for adolescents from urban and periurban settings of Uganda that reported high mean DMFT score values. Some reports [26, 35, 36] have indicated that students in urban areas of Uganda experience poor oral health outcomes due to the high cost of dental care, ignorance of established preventive oral health practices, and shortage of dental professionals.

It is noteworthy that restorative treatment was the main dental care needed by the students and tooth extractions increased with age (Table 5). Assuming that the rate of progression of caries takes 3–4 years before it gets to pulpitis requiring extraction, it is probable that for most of the participants, the initial tooth decay occurred in the early adolescent years (10–12 years). It would therefore be beneficial to target the adolescents aged 10–12 years with preventive oral health programmes. Decayed teeth (DT) were significantly associated with difficulty in chewing, history of dental pain in the past 12 months, poor perception of tooth state, and the female gender (Table 3), which corroborates with other studies [37, 38]. Due to these negative consequences, several reports [37, 39, 40] have documented the negative impact of decayed teeth on the quality of life, general health, nutrition, growth, and body weight of the affected individuals.

## 5. Conclusion and Recommendation

The prevalence of decayed teeth was high in the present study, and in order to satisfy the oral health needs of the population, it is recommended that health programmes in schools should include both oral health preventive and curative interventions to achieve optimum health.

## 6. Strengths and Limitations

The study provided baseline data on dental caries treatment needs for the Ugandan adolescent population. This study was carried out in field conditions, without use of radiographs or artificial light, which made it likely that the prevalence of decayed teeth and hence treatment needs could have been underestimated. The findings of the present study may not be generalised to all adolescents in Uganda due to the limited sample of schools and adolescents drawn from one region of Uganda.

## Figures and Tables

**Table 1:** The frequency distribution of study participants according to their characteristics (*n* = 406).

Variable	Categories	Frequency	Percentage
Age	11–13	65	16.0
	14–16	179	44.1
	17–19	162	39.9

Gender	Male	157	38.7
	Female	249	61.3

Tooth brushing frequency	At least once a day	379	93.4
	Never or occasionally	27	6.6

Dental pain in the last 12 months	No	208	51.2
	Yes	198	48.8

Chewing problem	No	235	57.9
	Yes	171	42.1

Perception of tooth status	Good	177	43.6
	Average	173	42.6
	Poor	56	13.8

Mother's highest education (*n* = 366)	Primary or below	93	25.4
	Secondary	197	53.8
	Tertiary	76	20.8

**Table 2:** Distribution of decayed teeth according to gender among the students.

Variable	Categories	Male, *N* (%)	Female, *N* (%)	Total, *n* (%)	*p* value
Decayed teeth (*n* = 406)	No	69 (43.9)	83 (33.3)	152 (37.4)	0.031
	Yes	88 (56.1)	166 (66.7)	254 (62.6)	

Number of decayed teeth (*n* = 254)	1	29 (33.0)	65 (39.2)	94 (37.0)	0.806
	2–3	34 (38.6)	59 (35.5)	93 (36.6)	
	4–6	20 (22.7)	33 (19.9)	53 (20.9)	
	7–8	5 (5.7)	9 (5.4)	14 (5.5)	

*n*: number; *p* values: chi-square test.

**Table 3:** Bivariate and multivariate Poisson regression analysis of decayed teeth (DT) and independent variables among participants (*N* = 406).

Variable	DT	Unadjusted		Adjusted			
	Mean (SD)	Rate ratio	95% CI	^*∗*^ *p* value	Rate ratio	95% CI	^*∗*^ *p* value
Gender							
Female	1.79 (2.32)	1	1	1	1	1	1
Male	1.60 (2.13)	0.89	0.77–1.04	0.150	0.83	0.71–0 .97	0.017
Frequency of cleaning teeth							
At least once a day	0.72 (2.26)	1	1	1	—	—	—
Never/occasionally	1.67 (2.04)	0.97	0.72–1.31	0.837	—	—	—
Perception of tooth status							
Good	1.31 (1.56)	1	1	1	1	1	1
Average	1.82 (2.29)	1.39	1.17–1.65	<0.001	1.05	0.88–1.26	0.606
Poor	2.70 (3.38)	2.06	1.68–2.53	<0.001	1.46	1.17–1.82	0.001
Dental pain in the past 12 months							
No	1.09 (1.43)	1	1	1	1	1	1
Yes	2.38 (2.72)	2.19	1.87–2.57	<0.001	1.87	1.55–2.26	0.000
Difficulty in chewing							
No	1.30 (1.75)	1	1	1	1	1	1
Yes	2.29 (2.69)	1.77	1.52–2.05	<0.001	1.23	1.03–1.46	0.022

Rate ratio: the DT ratio of each group versus reference group; *n*: number; CI: confidence interval; ^*∗*^*p* values: Poisson analysis; 1: reference value.

**Table 4:** The frequency distribution of the participants according to treatment needs, gender, and age group (*n* = 406).

Variable		Specific treatment need				
		Caries preventive treatment	Traumatic dental injuries	Decayed teeth		
		Fluoride application, *n* (%)	Fillings, *n* (%)	Filling, *n* (%)	Pulp therapy, *n* (%)	Extraction, *n* (%)
Overall	*N* = 406	17 (4.2)	11 (2.7)	241 (59.4)	54 (13.3)	37 (9.1)
Gender	Male (*n* = 157)	7 (4.5)	7 (4.5)	84 (53.5)	18 (11.46)	12 (7.6)
	Female (*n* = 249)	10 (4.0)	4 (1.6)	157 (63.1)	36 (14.5)	25 (10.4)
Age (in years)	11–13 (*n* = 65)	0 (0.0)	1 (1.5)	27 (41.5)	3 (4.6)	2 (3.1)
	14–16 (*n* = 179)	12 (6.7)	7 (3.9)	99 (55.3)	23 (12.8)	17 (9.5)
	17–19 (*n* = 162)	5 (3.1)	3 (1.9)	115 (71.0)	28 (17.3)	18 (11.1)

*n*: number; cross-tabulations of treatment needs by variables.

**Table 5:** The frequency distribution of the decayed teeth according to specific treatment needs, gender, and age group (*n* = 696).

Variable		Specific treatment needs for decayed teeth			
		Filling, *n* (%)	Pulp therapy, *n* (%)	Extraction, *n* (%)	Total, *n*
Overall		579 (83.2)	73 (10.5)	44 (6.3)	696
Gender	Male	216 (86.0)	22 (8.8)	13 (5.2)	251
	Female	363 (81.6)	51 (11.5)	31 (6.9)	445
Age (in years)	11–13	50 (90.9)	3 (5.5)	2 (3.6)	55
	14–16	231 (80.8)	34 (11.9)	21 (7.3)	286
	17–19	298 (83.9)	36 (10.2)	21 (5.9)	355

*n*: number; cross-tabulations of treatment needs for decayed teeth by gender.

## Data Availability

The data used to support the findings of this study are available from the corresponding author upon request.
